# Crystal structure of Middle East respiratory syndrome coronavirus helicase

**DOI:** 10.1371/journal.ppat.1006474

**Published:** 2017-06-26

**Authors:** Wei Hao, Justyna Aleksandra Wojdyla, Rong Zhao, Ruiyun Han, Rajat Das, Ivan Zlatev, Muthiah Manoharan, Meitian Wang, Sheng Cui

**Affiliations:** 1 MOH key Laboratory of Systems Biology of Pathogens, Institute of Pathogen Biology, Chinese Academy of Medical Sciences & Peking Union Medical College, No.9 Dong Dan San Tiao, Beijing, China; 2 Swiss Light Source at Paul Scherrer Institute, Villigen, Switzerland; 3 Alnylam Pharmaceuticals, Cambridge, MA, United States of America; Washington University, UNITED STATES

## Abstract

Middle East respiratory syndrome coronavirus (MERS-CoV) remains a threat to public health worldwide; however, effective vaccine or drug against CoVs remains unavailable. CoV helicase is one of the three evolutionary most conserved proteins in nidoviruses, thus making it an important target for drug development. We report here the first structure of full-length coronavirus helicase, MERS-CoV nsp13. MERS-CoV helicase has multiple domains, including an N-terminal Cys/His rich domain (CH) with three zinc atoms, a beta-barrel domain and a C-terminal SF1 helicase core with two RecA-like subdomains. Our structural analyses show that while the domain organization of nsp13 is conserved throughout nidoviruses, the individual domains of nsp13 are closely related to the equivalent eukaryotic domains of Upf1 helicases. The most distinctive feature differentiating CoV helicases from eukaryotic Upf1 helicases is the interaction between CH domain and helicase core.

## Author summary

Severe acute respiratory syndrome coronavirus (SARS-CoV) and Middle East respiratory syndrome coronavirus (MERS-CoV) caused global pandemics in 2003 and 2012 with the fatality rates of 10–35%. Outbreak of MERS-CoV in the Republic of Korea in 2015 highlighted that the newly emerged CoVs remain a concern for the public health. Nevertheless, effective vaccine and drug against CoVs are still missing. Among CoV-encoded nonstructural proteins (nsps), nsp13 helicase is considered one of the most important drug targets. Nsp13 is a highly conserved protein in CoVs and nidovirales and one of the two central components of the membrane associated replication-transcription complex, which performs viral RNA synthesis. However, despite decades of structural characterization of CoV-encoded proteins, the structure of nsp13 remained unavailable. In this study, we determined the first crystal structure of the full-length MERS-CoV nsp13. MERS-CoV helicase has an N-terminal Cys/His rich domain (CH) with three zincs, a beta-barrel domain and a C-terminal SF1 helicase core. While the domain organization of nsp13 is similar to arterivirus nsp10, the individual domains of nsp13 are closely related to their equivalent domains of eukaryotic Upf1 helicases. Our findings provide novel structural information essential for structure-based drug design against CoV.

## Introduction

Severe acute respiratory syndrome coronavirus (SARS-CoV) and Middle East respiratory syndrome coronavirus (MERS-CoV) caused global pandemics in 2003[1] and 2012[2] with the fatality rates of 10–35%. Outbreak of MERS-CoV in the Republic of Korea[3] in 2015 highlighted that the newly emerged CoVs remain a major concern for the public health. Nevertheless, effective vaccine and drug against CoVs are still missing.

MERS-CoV is a member of *Coronaviridae* family, one of the four distantly related virus families (the other three are *Arteriviridae*, *Mesoniviridae* and *Roniviridae*) in Nidovirales order[4–6]. This monophyletic group of viruses includes the largest known RNA genomes in families *Roniviridae* (~26 kb) and *Coronaviridae* (from 26.3 to 31.7 kb), as well as, small-sized *Arteriviridae* (12.7 to 15.7 kb) and medium-sized *Mesoniviridae* (20.2 kb)[7,8]. *Coronaviridae* family is divided into *Torovirinae* and *Coronavirinae* subfamilies, with the latter consisting of α-CoVs, β-CoVs, δ-CoVs and γ-CoV genera[5]. Six human CoVs have been identified to date, including α-CoVs 229E-CoV and NL63-CoV and β-CoVs OC43-CoV and HKU1-CoV from lineage A, SARS-CoV from lineage B and MERS-CoV from lineage C[9–11].

MERS-CoV has a positive single-stranded RNA (+RNA) genome of ~30kb, one of the largest among +RNA viruses[4]. To support the efficient replication of its exceptionally large genome, MERS-CoV encodes two replicase polyproteins pp1a and pp1ab, which are proteolytically processed into 16 nonstructural proteins (nsps)[12,13]. The nsps assemble into the membrane-associated replication-transcription complexes (RTCs), which drive viral genome replication and translation. An RNA-dependent RNA polymerase (nsp12) and a helicase (nsp13) are central components of RTC[14,15].

It has been previously shown that +RNA viruses with genome larger than 7 kb encode helicases [16–18]. Helicases unwind DNA or RNA duplexes in an NTP hydrolysis dependent manner. They are classified into six superfamilies SF1-SF6 and participate in almost every aspect of nucleic acid metabolism[19]. Regardless of their functional diversity, helicases all contain core domains that hydrolyze NTPs. The enzymatic core is formed either by the tandem RecA-like domains within the same polypeptide chain (SF1-SF2 superfamilies) or between subunits of the functional oligomer of the helicase (SF3-SF6 superfamilies) [16]. The universal features of the RecA-like domain includes a Walker A motif forming the phosphate binding loop (P-loop), a Walker B motif coordinating magnesium and an “arginine finger” engaging γ-phosphate of ATP[18,20,21]. In addition to the core domains, helicases also have accessory domains or inserts with various functions, such as assisting the catalytic activity or the interacting with other protein partner [16,17,22].

Sequence conservation analysis indicates that CoV nsp13 belongs to SF1 superfamily, including Rep, UvrD, PcrA, RecD, Pif1, Dda, Upf1-like helicases and many +RNA virus helicases[18,23]. Nidovirus helicases share many structural features with the eukaryotic Upf1 helicase, a key factor in nonsense-mediated mRNA decay in cells[24,25]. Upf1 is a multi-domain protein comprising of an N-terminal Cys-His-rich domain (CH domain) coordinating three zinc atoms, a 1B domain with the β-barrel fold and a conserved SF1 helicase core with a 1C insert in the first RecA-like domain[24,26]. The crystal structure of nsp10 from equine arteritis virus (EAV) of *Arterovirus* genus is the first high-resolution structure of nidovirus helicase. EAV nsp10 has an N-terminal zinc-binding domain (ZBD) that is followed by the 1B and a SF1 helicase core, but it lacks the 1C insert. The ZBD of nsp10 coordinates two zinc ions by an N-terminal RING-like module and one zinc ion by a C-terminal treble-clef zinc finger. The 1B domain of nsp10 undergoes large conformational change upon substrate binding, and 1B together with the 1A and 2A domains of the helicase core form a channel that accommodates the single stranded nucleic acids. The CH domain of Upf1 mediates the binding with Upf2[26]. Similarly, the ZBD of nsp10 harbors a putative protein interaction surface, of which the binding partner remains to be identified[25]. The structural resemblance between Upf1 and EAV nsp10 suggests that nidovirus helicase may be involved in the posttranscriptional quality control of the viral RNAs.

CoV helicase is one of the three evolutionary most conserved proteins in nidoviruses[27], thus making it an important target for drug development[28]. Previous biochemical characterizations have shown that CoV nsp13 exhibits multiple enzymatic activities, which include hydrolysis of NTPs and dNTPs, unwinding of DNA and RNA duplexes with 5’-3’ directionality and the RNA 5’-triphosphatase activity[29,30]. Additionally, the RNA dependent RNA polymerase (RdRP, nsp12) of CoV physically interacts with nsp13 and enhances its unwinding activity[31]. Although the molecular mechanism underlying these activities and the role of nsp13 in viral RNA synthesis are poorly understood, mutagenesis studies have identified a collection of residues important for the activity of nidovirus helicase. Disruption of the zinc binding function of 229E-CoV nsp13 or EAV nsp10 by replacing the conserved Cys/His residues at ZBD or deleting the entire zinc binding domain interfere with the ATPase activity of the helicases. Moreover, the activity of nsp10 is not complemented by providing wild-type ZBD in trans[32]. These results suggest that ZBD of nidovirus helicase modulates the ATPase/helicase activity in cis. CoVs nsp13 is essential for virus replication. ATPase/helicase deficient mutations of nsp13 (either at the zinc-binding site or the Walker A motif) can lead to the abolition of CoV replication. The mouse hepatitis virus (MHV) M protein and nsp13 are required for efficient replication An A335V mutation in the helicase core of nsp13 causes the attenuation of MHV replication both *in vitro* and *in vivo* [33].

SARS- and MERS-CoV outbreaks boosted nearly fifteen years of structural studies on the CoV proteins. However, despite extensive efforts, three-dimensional structural characterization of nsp13, one of the most important CoV replication enzymes, remained absent.

## Results

### Biochemical characterization of the recombinant MERS-CoV nsp13

**Fig 1:**
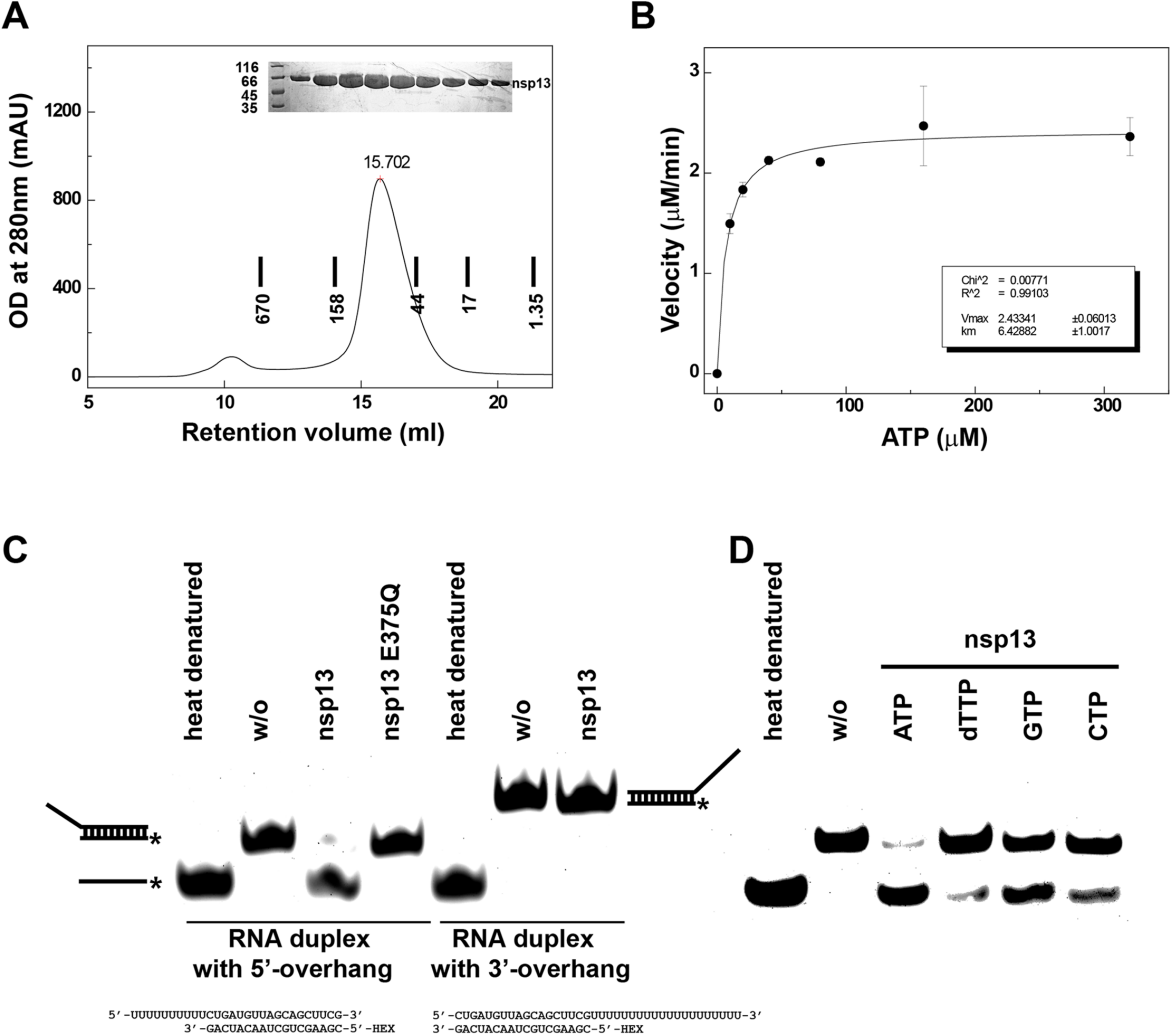
Enzymatic activities of recombinant MERS-CoV nsp13. MERS-CoV nsp13 carries NTPase activity and RNA duplex unwinding activity. (A) Final purification step of recombinant MERS-CoV nsp13 expressed in insect cells. MERS-CoV nsp13 eluted from Superdex 200 300/10 GL column pre-calibrated with gel filtration standards (thyroglobulin 670 kDa, γ-globulin 158 kDa, ovalbumin 44 kDa and myoglobin 17 kDa and vitamin B12 1,35 kDa). Upper insert, SDS-PAGE analysis of the purified protein. (B) ATPase activity of MERS-CoV nsp13. The velocity of ATP hydrolysis is plotted as the function of ATP concentration. The data was fitted to Michaelis–Menten equation to calculate *V*_*max*_ and *K*_*m*_. (C) Left, helicase assay shows that MERS-CoV nsp13 can unwind RNA partial duplex with 5’ overhang, but cannot unwind RNA partial duplex with 3’ overhang. RNA strand with HEX label is marked with asterisk. Heat denatured and no enzyme (w/o) controls are indicated. (C) Right, helicase assay showing MERS-CoV nsp13 can utilize different NTPs and dNTP to separate RNA strands with a preference to ATP. The sequence of the RNA substrates is shown at the bottom.

To investigate the structure of nsp13, we overexpressed the full-length MERS-CoV nsp13 (1-598aa) in High-5 insect cells (Fig 1A). To verify that the recombinant protein is enzymatically active we first performed ATPase assay. The purified nsp13 exhibited ATPase activity with a turnover number (*k*_*cat*_) of 2.03 ±0.1 s^-1^ and the catalytic efficiency (*k*_*cat*_ *K*_*m*_^*-1*^) of 0.32 μM^-1^ s^-1^ (Fig 1B). The ATPase activity of MERS-CoV nsp13 is comparable with that reported for SARS nsp13[29]. Next, we assessed helicase activity of the recombinant nsp13. Partial RNA duplex containing 5’ overhang was fully unwound by the purified nsp13. By contrast, MERS-CoV nsp13 could not unwind RNA duplex containing 3’ overhang (the duplex region remained the same as the RNA duplex with 5’ overhang). These results confirm that MERS-CoV nsp13 is a unidirectional helicase with the unwinding polarity of 5’-to-3’ (Fig 1C, left). Mutant with E375Q within Walker B failed to unwind the RNA substrate with 5’ overhang clearly indicating that the helicase activity of MERS-CoV nsp13 is dependent on ATP hydrolysis (Fig 1C, left). MERS-CoV nsp13 was able to hydrolyze different NTPs and dNTPs to support the unwinding of RNA substrate, with a clear preference towards ATP (Fig 1C, right). Our results are consistent with the recent enzymatic characterization of the MERS-CoV nsp13 expressed in bacteria[30].

### Structure determination of MERS-CoV nsp13

Our crystallization trials with the unliganded MERS-CoV nsp13 yielded crystal, which diffracted the X-rays poorly. Intriguingly, incubation with a 5’-triphosphate-15T DNA (ppp-15T) greatly improved diffracting power of nsp13 crystals. Benefiting from the presence of an N-terminal zinc-binding domain, we collected highly redundant multi-wavelength anomalous diffraction (MAD) data at the zinc absorption edge and subsequently solved the structure. Next, we collected a 3.0Å resolution native dataset, which we used for final structure refinement and further analysis. There are two nsp13 in the asymmetric unit (ASU) with three zinc ions bound to each N-terminal Cys/His rich domain. Unexpectedly, no additional electron density for ppp-15T DNA could be identified, indicating that a stable nsp13-DNA complex did not form. The final model of nsp13 has good stereochemistry quality. Data collection and refinement statistics are summarized in Table 1.

**Table 1:** Data collection and refinement statistics.

	MERS-CoV nsp13			MERS-CoV nsp13 (PDB ID: 5WWP)
**Data collection**	**MAD phasing**			**native data**
Space group	P6_1_22			P6_1_22
Cell dimensions				
a, b, c (Å)	186.19 186.19 185.44			185.68 185.68 185.09
α, β, γ (°)	90.00 90.00 120.00			90.00 90.00 120.00
X ray source	SLS X06DA			SSRF BL19U1
Wavelength (Å)	1.2827 (Peak)	1.2831 (Infl)	1.2810 (Hrem)	0.9784 (native)
Data range (Å)	49.06–3.12	49.09–3.12	49.05–3.12	48.95–3.00
Reflections unique	63859	64027	63771	71207
*R*_sym_ ^a^ (last shell)	0.29 (1.89)	0.29 (2.01)	0.30 (1.98)	0.08 (0.75)
CC(1/2)	99.7 (61.6)	99.7 (59.6)	99.3 (47.7)	99.8 (57.1)
*I* / σ*I*	13.80 (1.76)	13.64 (1.67)	10.66 (1.31)	12.27 (1.57)
Completeness (%) (last shell)	99.9 (99.7)	99.9 (99.8)	99.9 (99.7)	99.4 (98.4)
Redundancy (last shell)	21.15 (19.79)	21.14 (19.77)	12.91 (12.08)	3.38 (3.36)
**Refinement**				
Resolution range (Å)				48.95–3.00
% reflections in cross validation				4.81
*R*_work_ ^b^/ *R*_free_ ^c^ (last shell)				0.23, 0.28 (0.38, 0.41)
**Atoms**				
All atoms				8571
Protein				8540
Zinc				6
Solvent				25
*B*-factors average (Å^2^)				68.1
Protein (Å^2^)				68.1
Ligands (Å^2^)				68.5
Solvent (Å^2^)				90.2
**r.m.s.d**				
Bond lengths (Å)				0.015
Bond angles (°)				0.950
**Validation**				
MolProbity score				2.75, 88^th^ percentile ^d^
Clashscore, all atoms				15.39, 97^th^ percentile ^d^
% residues in favored regions, allowed regions, outliers in Ramachandran plot				91.8, 7.5, 0.7

^a^ *R*_sym_ = ∑_hkl_∑_j_ |Ihkl,j—I_hkl_|/∑_hkl_∑_j_I_hkl,j_, where I_hkl_ is the average of symmetry-related observations of a unique reflection

^b^ *R*_work_ = ∑_hkl_ ||*F*_obs_(hkl)|-|*F*_calc_(hkl)||/∑_hkl_|*F*_obs_(hkl)|.

^c^ *R*_free_ = the cross-validation *R* factor for 5% of reflections against which the model was not refined.

^d^ 100^th^ percentile is the best among structures of comparable resolution; 0^th^ percentile is the worst. For clashscore the comparative set of structures was selected in 2004, for MolProbity score in 2006.

### Overall domain organization of MERS-CoV nsp13

**Fig 2:**
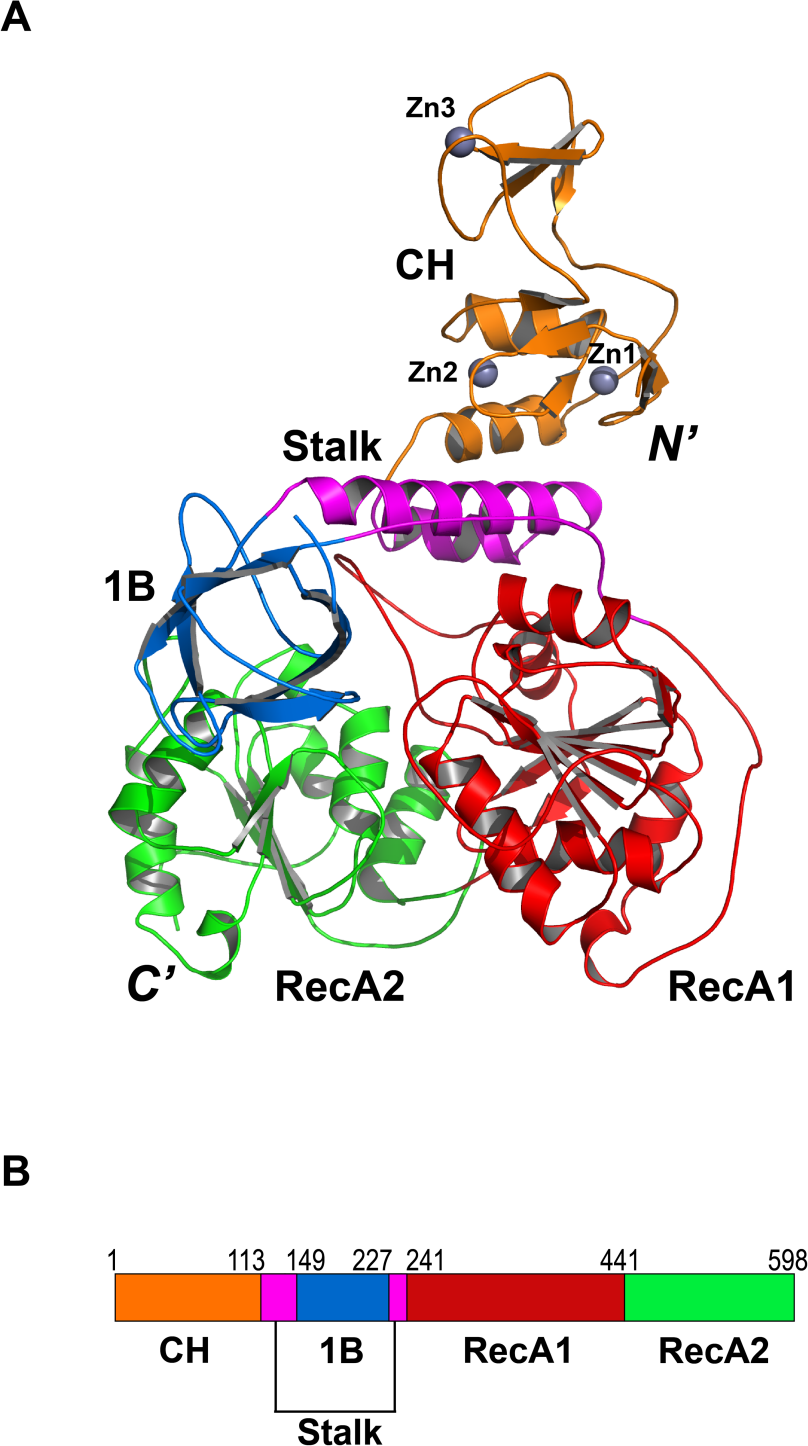
Overall structure of MERS-CoV nsp13. (A) Ribbon model of MERS-CoV nsp13 containing CH (orange), Stalk (magenta), 1B (blue), RecA1 (red) and RecA2 (green) domains. (B) Schematic diagram of the domain organization of MERS-CoV nsp13.

**Fig 3:**
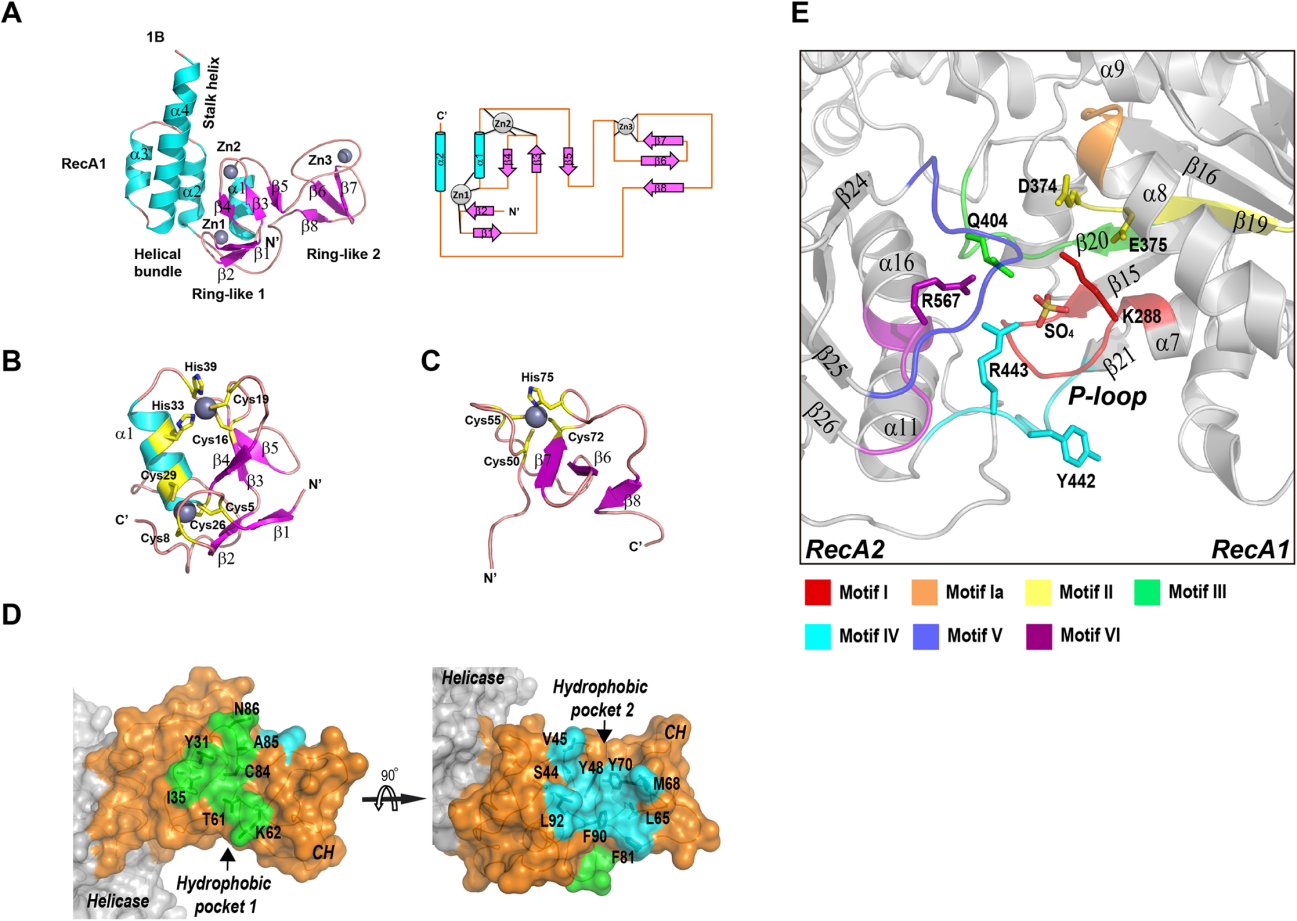
Key structural features of MERS-CoV nsp13. (A) left, ribbon model of the CH and Stalk domains of MERS-CoV nsp13 colored by secondary structural elements; right, 2-D topology graph of the CH domain. (B) ribbon model of N-terminal Ring module, and (C) C-terminal Ring module of the CH domain. His/Cys residues participating in zinc coordination are highlighted in yellow. (D) Surface representation of CH domain (orange) of MERS-CoV nsp13, two hydrophobic pockets equivalent to protein interaction interfaces on Upf1 are highlighted in green (pocket 1) and cyan (pocket 2). (E) The ATPase active site between RecA1 and RecA2 domains. The conserved helicase motifs are highlighted with different colors.

**Fig 4:**
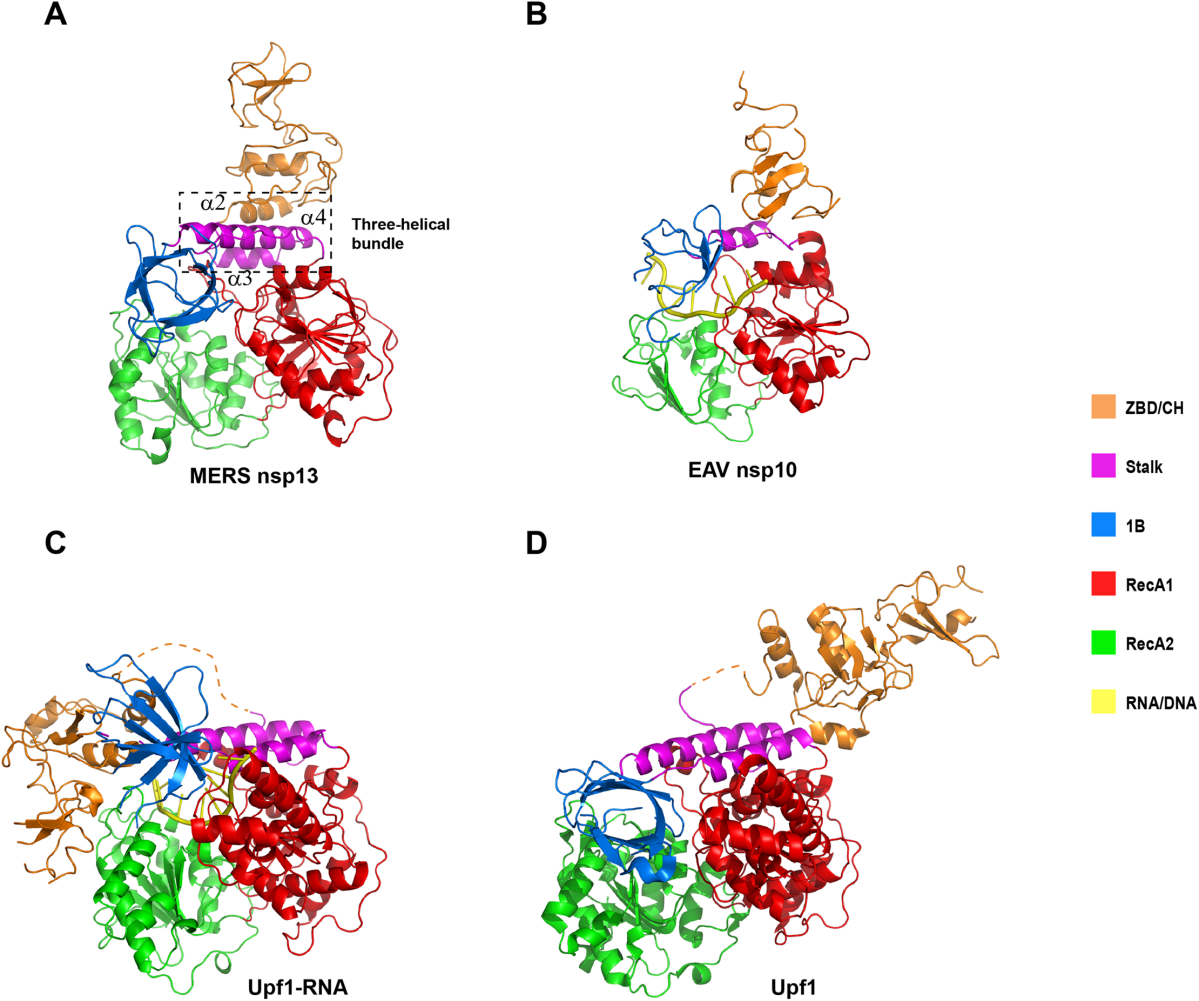
Structural comparison of Upf1-related helicases. MERS-CoV nsp13 is colored in cyan, EAV nsp10 is colored in blue and Upf1 is colored in magenta. Secondary structure elements or subdomains of MERS-CoV nsp13 are labeled with regular font, secondary structure elements of EAV nsp10 and Upf1 helicase are labeled with italic font. (A) The structure of CH of Upf1 (PDB ID: 2XZL) is superimposed with the CH of MERS-CoV nsp13 (PDB ID: 5WWP). Three zinc atoms of each CH domain are indicated. (B) The structure of ZBD of EAV nsp10 (PDB ID: 4N0N) is superimposed to the CH domain of MERS-CoV nsp13. Three zinc atoms of each CH or ZBD are indicated (italic fonts). (C) Structure based multiple sequence alignment of CH/ZBD domains of Upf1 like helicases, including helicases from human CoVs (MERS-CoV, SARS-CoV, HKU1-CoV, OC43-CoV, NL63-CoV and 229E-CoV), EAV nsp10 and Upf1 helicase. The sequences were aligned using MUSCLE software [45]. Cys/His residues involved in the coordination of Zn1, Zn2 and Zn3 are colored with magenta, blue and cyan, respectively. The secondary structure elements are aligned to the sequence. (D) Superimposition of MERS-CoV nsp13 and Upf1. (E) Superimposition of MERS-CoV nsp13 and EAV nsp10. MERS-CoV nsp13 is the reference structure with the same orientation as in Fig 2A. The subdomains of the helicases are labeled (regular fonts for MERS-CoV nsp13, italic fonts for Upf1 or EAV nsp10).

MERS-CoV nsp13 is composed of multiple functional domains (Fig 2, S1 and S2 Figs). The N-terminal CH domain has 15 conserved Cys/His residues, twelve of which participate in the coordination of three zinc ions (Fig 3A & Fig 4A–4C). The C-terminal helicase belongs to the SF1 helicase family and consists of two “RecA-like” domains, referred to as RecA1 and RecA2. The CH and helicase are connected *via* a region consisting two additional domains. A helical domain sandwiched between the CH and RecA1 domains is followed by a six-stranded anti-parallel β-barrel domain. Because of the structural resemblance to the domains of Upf1 helicases, these domains were named as Stalk and 1B, respectively. We compared the crystal structure of MERS-CoV nsp13 with all structures in Protein Data Bank using Dali server. The top hits were human Upf1 helicase[24] (hUpf1 PDB code: 2GK7; Dali Z-score: 22.7) and arterivirus (EAV) helicase[25] (PDB code: 4N0N; Dali Z-score: 22.2). The structural superposition showed that while the helicase portion of these three proteins aligns well, the N-terminal CH (or ZBD) and 1B domains adopt various conformations relative to the helicase core.

### Structure of the CH domain of MERS-CoV nsp13

The CH domain of MERS-CoV nsp13 (residues 1–112) is a compact domain with three zinc-binding motifs stabilizing the fold (Fig 3A). The CH domain contains an N-terminal RING-like module (1-46aa, β1–5 and α1) with two zinc fingers and C-terminal RING-like module with single treble-clef zinc finger (47-87aa, β6–8). A long loop (88-100aa, between β8-α2) spanning across the entire height of CH domain connects zinc coordination modules. The first zinc (Zn1) is coordinated within a CCCC type treble-clef zinc finger with four cysteine ligands (Cys5, Cys8 Cys26 and Cys29) (Fig 3B). Cys5 and Cys8 are located on a zinc knuckle between β1-β2 strands, whereas Cys26 and Cys29 are placed in the N-terminal region of α1 helix. The second zinc (Zn2) is coordinated by a two-Cys, two-His (C2H2) zinc finger motif. The cysteine ligands (Cys16 and Cys19) are forming part of a zinc knuckle between β3-β4 strands, while histidine ligands His33 and His39 are located on α1 helix and the loop between α1 and β5, respectively. The third zinc (Zn3) is coordinated by the C-terminal treble-clef zinc finger motif (Fig 3C). A zinc-knuckle on a hairpin loop between β5 and β6 strands provides Cys50 and Cys55 for Zn3 coordination, whereas Cys72 from the C-terminal region of β7 strand and His75 from the loop between β7 and β8 strands provide two more ligands for the zinc. All Cys/His involved in zinc coordination are invariant in CoVs (Fig 4C), indicating the zinc binding is essential for the structure and function of nsp13.

### Comparison of the CH/ZBD of MERS-CoV nsp13, EAV nsp10 and Upf1

**Fig 5:**
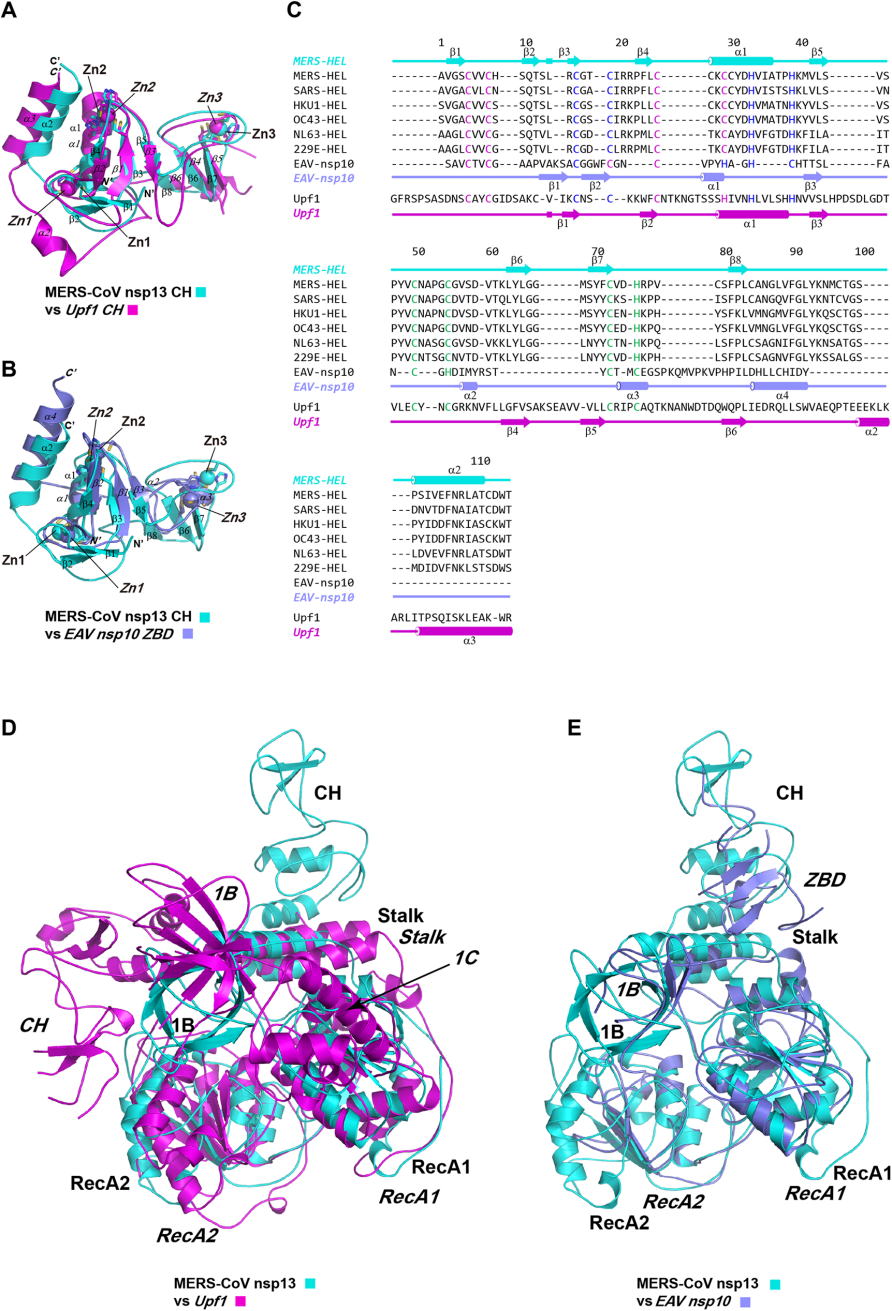
Domain composition of Upf1-related helicases. Ribbon models of (A) MERS-CoV nsp13, (B) EAV nsp10-DNA PDB ID: 4N0O, (C) Yeast Upf1-RNA PDB ID: 2XZL and (D) Human Upf1 without RNA PDB ID: 2WJV colored by different functional domains. The coloring scheme is indicated on the right.

We compared the isolated CH/ZBD of MERS-CoV nsp13, EAV nsp10 and Upf1 (Table 2), and found that the CH of MERS-CoV nsp13 is structurally more related to the CH of Upf1 than to the ZBD of EAV nsp10. Superimposition of yeast Upf1 CH (scUpf1 PDB code: 2XZL) and MERS-CoV nsp13 CH aligned 102 Cα atoms with the Dali Z-score of 10.2 and rmsd of 2.2Å. Superimposition of EAV nsp10 CH (PDB code: 4N0N) and MERS-CoV nsp13 CH aligned 59 Cα atoms with the Dali Z-score of 4.4 and rmsd of 3.4Å. Additionally, while the positions of three zincs of MERS-CoV nsp13 CH domain and Upf1 CH domain are nearly identical, Zn3 of EAV nsp10 ZBD is located much closer to Zn1 and Zn2. Particularly, the distance from Zn3 to Zn2 is ~6.4Å shorter in EAV nsp10 than the corresponding distance in MERS nsp13 (Fig 4B). However, the interaction between the CH domain and the helicase region is significantly different between nidovirus helicases and Upf1, and similar between MERS-CoV nsp13 and EAV nsp10. The MERS-CoV nsp13 CH domain is tightly attached to the Stalk domain (Fig 5A). Helices α2-α4 form a three-helical bundle. The buried area between CH and Stalk is 1670Å^2^. Similarly, the EAV nsp10 ZBD domain is connected to the stalk domain, which is significantly smaller than CH of MERS-CoV nsp13 (Fig 5B). The buried area between ZBD and Stalk is 634.7 Å^2^. By contrast, the CH of Upf1 helicase is linked to the helicase portion through a long and flexible loop (often invisible in the electron density maps), allowing large movement of the CH domain (Fig 5C and 5D)[26].

**Table 2:** Pairwise comparison of the isolated CH/ZBD, 1B and helicase core domains of MERS-CoV nsp13, EAV nsp10 and Upf1 helicases.

Domain	Comparison	*Dali Z-score*	*rmsd(Å)*
**CH/ZBD**	nsp13 vs nsp10	4.4	3.4
	nsp13 vs Upf1	10.2	2.2
	nsp10 vs Upf1	3.0	3.5
**1B**	nsp13 vs nsp10	3.4	2.5
	nsp13 vs Upf1	2.8	3.3
	nsp10 vs Upf1	2.8	2.8
**Helicase** **Core**	nsp13 vs nsp10	23.2	2.7
	nsp13 vs Upf1	26.3	4.5
	nsp10 vs Upf1	20.9	3.6

nsp13: MERS-CoV nsp13, PDB ID: 5WWP

nsp10: EAV nsp10, PDB ID: 4N0N

Upf1: scUpf1 helicase, PDB ID: 2XZL

### Putative protein interaction surfaces on MERS-CoV nsp13 CH domain

Structural comparison of nsp13 CH and hUpf1 CH in complex with Upf2[34] revealed two hydrophobic pockets on the surface of nsp13 CH equivalent to Upf2 binding sites on Upf1 (Fig 3D). While pocket 1 highly resembles Upf2 α-helix binding site, the pocket 2 has a much shorter β6-β7 loop than the equivalent loop in Upf2 β-hairpin binding site of Upf1 (β5-β6 loop). Two hydrophobic pockets on CH domain may function as interaction interfaces for other CoV replicase or cellular protein.

### Structure of the nucleotide-binding pocket and the active site

The nucleotide-binding pocket of MERS-CoV nsp13 is located between RecA1 and RecA2 domains. The RecA1 (241-443aa) contains a seven-stranded parallel β-sheet sandwiched by two α-helices located near the Stalk domain on one side and three α-helices on the opposite side. RecA2 (444-596aa) has a five-stranded parallel β-sheet surrounded by four helices on one side and three helices on the other side. Seven helicase motifs conserved in SF1/SF2 families are located in the cleft between RecA1-RecA2. RecA1 contains motifs I, Ia, II and III, whereas RecA2 includes motifs IV, V and VI (Fig 3E). Sulfate, crystallization condition precipitant, was found bound to the P-loop mimicking binding of the NTP’s phosphate moiety. Residues Gln404, Arg443 and Arg567 from helicase motifs III, IV and VI form hydrogen bonds with the sulfate suggesting their involvement in NTP hydrolysis. The corresponding residues in human Upf1 helicase are Gln665, Arg703 and Arg865[24], while in EAV nsp10 Gln267, Arg296 and Arg381[25]. Residues Arg865 and Gln665 of Upf1 helicase act as the “arginine finger” and “γ-phosphate sensor” during ATP hydrolysis[24], suggesting that MERS-CoV nsp13 Gln404 and Arg567 have the same function. Moreover, Tyr442 of MERS-CoV nsp13 is structurally equivalent to Tyr702 of Upf1, which stabilizes adenosine base (Fig 3E).

### A model of MERS-CoV nsp13 in complex with single stranded RNA

**Fig 6:**
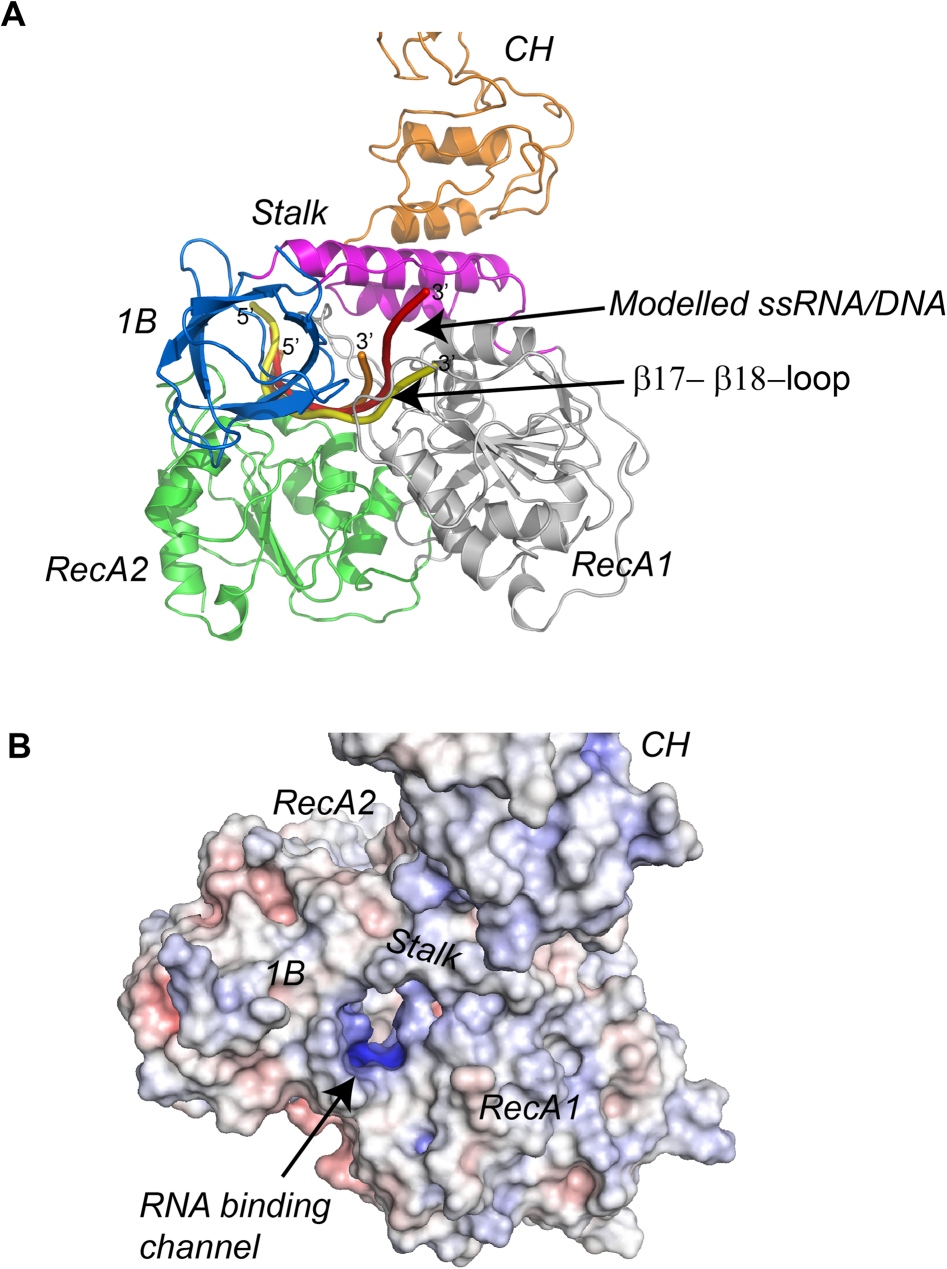
Model of RNA binding to MERS-CoV nsp13. **(A)** Model of MERS-CoV nsp13-ssRNA complex was generated based on the superimposition of the helicase domain on nsp13 with helicase domains of human Upf1-RNA (PDB ID: 2XZO, orange), yeast Upf1-RNA (PDB ID: 2XZL, red) and EAV -DNA (PDB ID: 4N0O, yellow). (B) Solvent accessible surface and electrostatic potentials of MERS-CoV nsp13. The functional domains and the putative nucleic acids binding channel are indicated.

It has been previously shown that CoV nsp13 interacts with both RNA and DNA in a sequence-independent manner[29,35,36]. To analyze the nucleic acid binding pocket of MERS-CoV nsp13, we generated a model of nsp13-ssRNA based on the superposition of the helicase domain of nsp13 with the helicase domains of hUpf1, scUpf1[26] and EAV nsp10[25]. The model shows that while the 3’ end of single-stranded RNA is located in the channel formed by 1B, Stalk and RecA1 domains; the 5’ end of the RNA lies on top of RecA2 domain (Fig 6A). Although the helical insertion equivalent to the Upf1 1C domain is missing in nsp13, the protein has a topologically equivalent loop between β17 and β18 which fulfills similar function in RNA binding. The β17-β18-loop makes direct contacts with 1B domain, forming the 3’ end outlet of the putative RNA binding channel. The narrowest opening of the putative RNA binding channel has the width and height of 6 and 12Å, which is just enough to accept a single-stranded RNA or DNA (Fig 6B). The dimension of the RNA binding channel of nsp13 is similar to that in EAV nsp10, but smaller than the channel in Upf1. Based on structural comparisons, we predicted the residues of nsp13, which are likely involved in RNA recognition (S2 Fig & S1 Table). Their structural equivalents are mostly conserved in EAV nsp10 and Upf1 helicases, suggesting that CoV nsp13 adopts the similar mechanism for nucleic acids binding.

## Discussion

Structural comparisons of the individual domains of Upf1-like helicases using Dali server showed that the CH of MERS-CoV nsp13 is structurally closer to the CH of Upf1 than to the ZBD of EAV nsp10. In accordance with previously published results, the structure of the helicase cores is well conserved in all three Upf1-like helicases (Table 2). However, some subtle differences could also be identified. Firstly, while the helicase cores of MERS-CoV nsp13 and Upf1 have similar size, the helicase core of EAV nsp10 is more compact. MERS-CoV nsp13 contains seven and five parallel β-strands in RecA1 and RecA2 domains, respectively, Upf1 has seven and six β-strands in the RecA1 and RecA2 domain, respectively, whereas EAV nsp10 has only five and four β-strands in the RecA like domains. Secondly, while three Upf1 like helicases all contain the 1B domain with the β-barrel fold, only Upf1 has a helical 1C insertion in RecA1 domain. The equivalent 1C insertion is missing in both EAV nsp10[25] and MERS-CoV nsp13 (Fig 4D and 4E). Thirdly, CoVs nsp13 does not contain a C-terminal domain homologous to the C-terminal portion of EAV nsp10 (C-terminal 65 residues), which was shown to regulate ATPase and helicase activities[25]. The sequence conservation analysis showed that the C-terminal domain of arteriviruses and CoVs is indeed poorly conserved[25]. Our crystallographic study provides the complete structure of the extreme C-terminal region of MERS-CoV nsp13, which shows that the C-terminus is an integral part of the RecA2 domain. Thus, we conclude that the C-terminal regulatory domain outside SF1 helicase core is completely missing in MERS-CoV nsp13 and other CoVs helicases.

Previous mutagenesis studies of CoVs nsp13 have identified a collection of residues essential to the activity of nsp13. Because nsp13 helicase is highly conserved among CoVs, the crystal structure of MERS-CoV nsp13 can provide three-dimensional information to understand previous phenotypes of nsp13 mutants. It has been shown that mutations of the conserved Cys/His at ZBD of 229E-CoV nsp13 interfered with its ATPase activity[32]. Ala or Arg replacement of C5003, C5021, C5024 and H5028 abolished or reduced the ATPase activity of 229E-CoV nsp13. Our crystal structure of MERS-CoV nsp13 confirms that these residues, corresponding to C8, C26, C29 and H33 of MERS-CoV nsp13 (Fig 4C) are coordinating Zn1 and Zn2. The loss of ATPase activity caused by Cys/His substitutes can be attributed to the disruption of zinc-binding and the integrity of the CH domain. Unexpectedly, only C5050A mutant retained significant ATPase activity (~60%). Based on the comparison with corresponding C55 of MERS-CoV nsp13, C5050 of 229E-CoV coordinates Zn3 of the ZBD. Zn3 is the most distantly located zinc from the helicase core (6.4Å away from Zn2; Fig 1A), and the ZBD/CH is tightly attached to the helicase core in CoV nsp13, it is therefore unsurprising that the impaired binding of Zn3 has the minimal effect on the enzymatic activity of nsp13[32]. This hypothesis is supported by the mutagenesis study of EAV nsp10[25,32], which also showed that while mutant H2414A (ligand for Zn3) retained residual ATPase/ helicase activity and wild-type-level nucleic acid binding activity, the activities of mutants C2395A (ligand for Zn1) and H2399A (ligand for Zn2) was completely abolished.

Recently, Zhang et al found that mutation A335V of MHV nsp13 led to attenuation of virus growth in cells and resulted in ~30 fold reduced viral titer in the livers of infected mice[33]. Residue A335 of MHV nsp13 is highly conserved among CoVs nsp13 (S2 Fig). Based on the crystal structure of MERS-CoV nsp13, A335 (corresponding to A336 of MERS-CoV nsp13) is located on the β17-β18-loop, a loop interacts with the 1B domain as well as participates in the formation of RNA binding channel. Because A335 is highly exposed to the surface of nsp13, Valine replacement could increase hydrophobicity of this region. Therefore, A335V mutation may impair the function of nsp13 by destabilizing the local structure critical to RNA binding or promoting aggregation of the protein.

In summary, our current study presents the first structural insight into the multiple functionality of the CoV nsp13. Our analyses demonstrate that while the domain organization of MERS-CoV nsp13 and EAV nsp10 is conserved, the structures of the individual domains of nsp13 are closely related to their eukaryotic equivalents in Upf1 helicase. While the interaction between the CH (or ZBD) domain and the helicase core presents the most distinctive feature differentiating nidovirus helicases from the Upf1 helicases, the high resemblance between the CH domains of MERS-CoV nsp13 and Upf1 helicases is remarkable. This structural similarity not only supports a hypothesis that nidoviruses helicase may have a Upf1-like role in post-transcriptional quality control of viral RNAs synthesis[25], but also implies a possibility that CoV nsp13 might use its Upf1-similar CH domain to interfere with nonsense-mediated mRNA decay (NMD) pathway. It has already been shown that NMD targets viral RNAs for degradation in the early phase of infection of +RNA viruses[37]. Based on the coevolution of virus and host mechanism, the viruses also develop strategies to suppress NMD. Nevertheless, whether CoV nsp13 is involved in NMD suppression requires experimental evidence. To investigate what proteins bind nsp13 through its CH domain and how do they modulate nsp13 function is important to expand our knowledge about the evolution and function of Upf1-like helicases. Finally, our results provide novel structural information essential for structure-based drug design against CoV.

## Materials and methods

### Protein expression, purification and crystallization

The gene encoding full-length MERS-CoV nsp13 helicase (1-598aa, GeneBank accession: YP_009047202) was amplified by polymerase chain reaction (PCR) and inserted via BamHI/XhoI restriction sites into a modified pFastbac-1 baculovirus transfer vector with an N-terminal 6×His-SUMO tag[38]. Nsp13 protein was overexpressed in High-5 insect cells using Bac-to-Bac Baculovirus Expression System (Invitrogen). One liter cell culture (2.0×10^6^ cells ml^-1^) was infected with 30 ml baculovirus at 22°C. Forty-eight hours after infection, cells were harvested by centrifugation. The cell pellet was re-suspended in a lysis buffer containing 25mM Tris-HCl (pH 7.5), 1.5 M NaCl and 15mM imidazole and lysed by ultrasonification. High salt concentration in the lysis buffer was necessary to remove nucleic acids bound to nsp13. Protein was purified using Ni-NTA resin (QIAGEN). The eluted protein was digested with PreScission protease (GE healthcare) to remove the 6×His-SUMO tag. Finally, the untagged nsp13 was purified using size-exclusion chromatography (Superdex-200, GE healthcare). The purified nsp13 was concentrated to ~8 mg/ml in the buffer containing 10mM HEPES (pH 7.0) and 100mM NaCl. Before crystallization trials, nsp13 was mixed with 5’-triphosphate 15 thymine single-stranded DNA (ppp-15T) with 1:1.5 molar ratio and incubated at 4°C overnight. Crystallization of nsp13 was achieved by mixing equal volume of sample and reservoir buffer (1.0μl) containing 0.1 M Tris-HCl (pH 8.5), 1M (NH_4_)_2_SO_4_ and 15% glycerol. The crystals were grown in a hanging-drop vapor-diffusion system at 18°C.

### Oligonucleotide synthesis

5’-triphosphate DNA (ppp-15T) was synthesized and purified according to previously published procedures[39,40]. Briefly, ppp-15T oligonucleotide was analyzed by LC/ESI-MS on a XBridge C8 column (2.1 x 50 mm, 2.5μm). Buffer A was 95mM 1,1,1,3,3,3-hexafluoro-2-propanol, 16mM triethylamine in water and buffer B was 100% methanol. A gradient from 2% to 29% B over 26.8 min with flow rate of 0.25 mL/min was employed at a column temperature of 60°C. A DNAPac-200 column (4 x 250 mm) was used for analytical IE-HPLC. Buffer A was 25mM Tris-HCl, 1mM EDTA in 10% acetronitrile (pH = 8) and buffer B was buffer A plus 1 M sodium bromide. A gradient of 25 to 56% B over 21.5 min at a flow rate of 1.0 mL/min was used at a column temperature of 75°C. Final purity by IE-HPLC and LC/ESI-MS was above 94% (S3 Fig).

### Structure determination

Prior to crystallization trials nsp13 protein was incubated with a 5’-triphosphate single-stranded DNA containing 15T (ppp-15T). Highly redundant multi-wavelength anomalous diffraction data were collected using the X-ray with wavelengths close to the absorption edge of zinc (Hrem: 1.2810Å, Peak: 1.2827Å and Infl: 1.2831Å). Crystal belonged to the space group P6_1_22, and contained two molecules per ASU. An interpretable electron density map was calculated using SHARP/autoSHARP[41]. An initial model of MERS-CoV nsp13 was manually built using Coot[42]. Finally, a native data with highest resolution (3.0Å) was collected using the X-rays with the wavelength of 0.978Å. Higher resolution structure was solved by molecular replacement using the initial nsp13 structure as the searching model. The 3.0Å structure was refined using PHENIX[43]. In the final model, 145-230aa (the entire 1B domain) of molecule A are disordered, probably due to mobility of 1B and the lack of crystal contacts, whereas in molecule B, 591 out of 598 amino acids were located in the electron density maps (S4 Fig).

### ATPase assay

ATPase assay was carried out as previously described[44]. Briefly, reaction mixtures (50μl) containing 100mM Tris-HCl (pH 8.0), 4mM MgCl_2_, trace amount of [γ-^32^P]ATP (~1nM) and the specified amount of ATP (from 10μM to 320μM) were incubated at 30°C. The reaction was initiated by the addition of MERS-CoV nsp13 (20nM). At each indicated time point, 2μl of quenching buffer (0.5M EDTA) was added to the mixture to stop the reaction. Finally, 1μl the sample was spotted on the thin-layer chromatography cellulose TLC plates (Sigma-Aldrich) and resolved with running buffer containing 0.8M acetate and 0.8M LiCl. The plates were dried and analyzed using storage phosphor screen and Typhoon Trio Variable Mode Imager (GE healthcare). ATP turnover was quantified using ImageQuant TL software (GE Healthcare).

### Helicase assay

The reaction mixture (10μl) contained 50mM HEPES (pH 7.5), 5mM MgCl_2_, 2mM dithiothreitol (DTT), 1mM nucleotide (ATP, GTP, CTP or TTP), 50nM of partial duplex RNA substrate and 300nM unlabeled trap RNA (5’-CGAAGCUGCUAACAUCAG-3’)[36]. The RNA substrate with 5’ overhang is prepared by mixing a top strand: 5’-UUUUUUUUUUCUGAUGUUAGCAGCUUCG-3’ with a bottom strand: 5’-HEX-CGAAGCUGCUAACAUCAG-3’. The RNA substrate with 3’ overhang is prepared by mixing a top strand: 5’-CUGAUGUUAGCAGCUUCGUUUUUUUUUUUUUUUUUUUU-3’ with the same bottom strand as in RNA partial duplex with 5’ overhang. The reaction was initiated by the addition of MERS-CoV nsp13 (100nM) and incubated at 30°C for 30 minutes. The reaction was terminated by addition of 2.5μl loading buffer (5X) containing 100 mM Tris-HCl (pH7.5), 1% SDS, 50mM EDTA and 50% glycerol. Samples were resolved by 10% native-PAGE running on ice. The gel was scanned with Typhoon Trio Variable Mode Imager (GE healthcare).

### Sequence and structure alignments

Multiple sequence alignment was carried out using MUSCLE software (http://www.ebi.ac.uk/Tools/msa/muscle/). In some region, minor manual adjustments were performed in accordance to the structural superimposition of the Upf1 like helicases. The illustration of sequence alignment was either generated using ESPript 3.0 (http://espript.ibcp.fr/ESPript/ESPript/), or produced manually using Microsoft PowerPoint. The structural alignment was carried out using Dali server (http://ekhidna.biocenter.helsinki.fi/dali_server).

## Supporting information

S1 Fig Stereo image of full-length MERS-CoV nsp13. A wall-eye stereo image of a ribbon model of MERS-CoV nsp13. The color scheme is the same as in Fig 2. Zinc atoms are shown with gray spheres.
(JPG)

S2 Fig Structure-based multiple sequence alignment of helicase core of nsp13 from different CoVs and EAV nsp10. Structure-based multiple sequence alignment of helicase core of nsp13 from human coronavirus (MERS-CoV, SARS-CoV, HKU1-CoV, OC43-CoV, NL63-CoV and 229E-CoV) and EAV nsp10. Sequence alignment of the CH/ZBD domains of CoV nsp13 and EAV nsp10 is shown in Fig 4C. Invariant residues are highlighted with red background; conserved residues are in red. Secondary structure elements are aligned to the top of the sequences. Conserved helicase motifs are indicated at the bottom of the sequences. Multiple sequence alignments were carried out using the program MUSCLE [45]. The program ESPript v3.0 was used to generate the figure[46].
(TIF)

S3 Fig Analytical data for compound ppp-15T. Compound: 5’-PPP-dTdTdTdTdTdTdTdTdTdTdTdTdTdTdT-3’ Calc MW: 4739.64, Found: 4739.89.
(TIF)

S4 Fig Portion of the electron density map of MERS-CoV nsp13 crystal structure. A wall-eye stereo image of a portion of electron density map (zn3 binding site). 2Fo-Fc map is shown with blue mesh. The final model of MERS-CoV nsp13 (green) is superimposed. The zinc is shown with a gray sphere.
(JPG)

S1 Table Prediction of residues of nsp13 involving in RNA recognition. (DOCX)
