# Supplementary figures and images for: Crystal structure of Middle East respiratory syndrome coronavirus helicase
Source: PLoS Pathog. 2017 Jun 26;13(6):e1006474. doi: 10.1371/journal.ppat.1006474 (PMC5501694; doi:10.1371/journal.ppat.1006474)

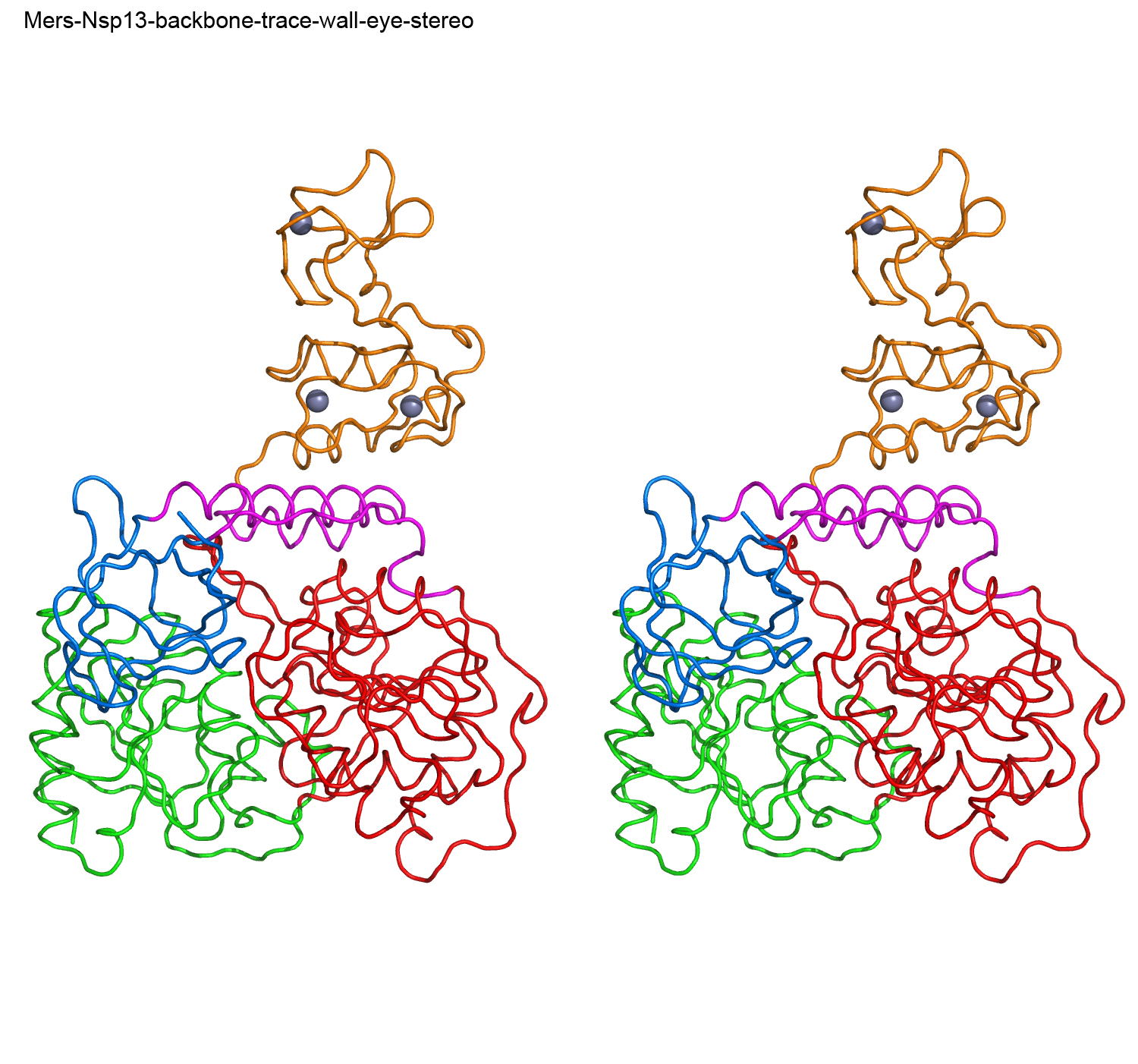

Supplement: S1 Fig — A wall-eye stereo image of a ribbon model of MERS-CoV nsp13. The color scheme is the same as in Fig 2. Zinc atoms are shown with gray spheres. (JPG) [file ppat.1006474.s001.jpg]

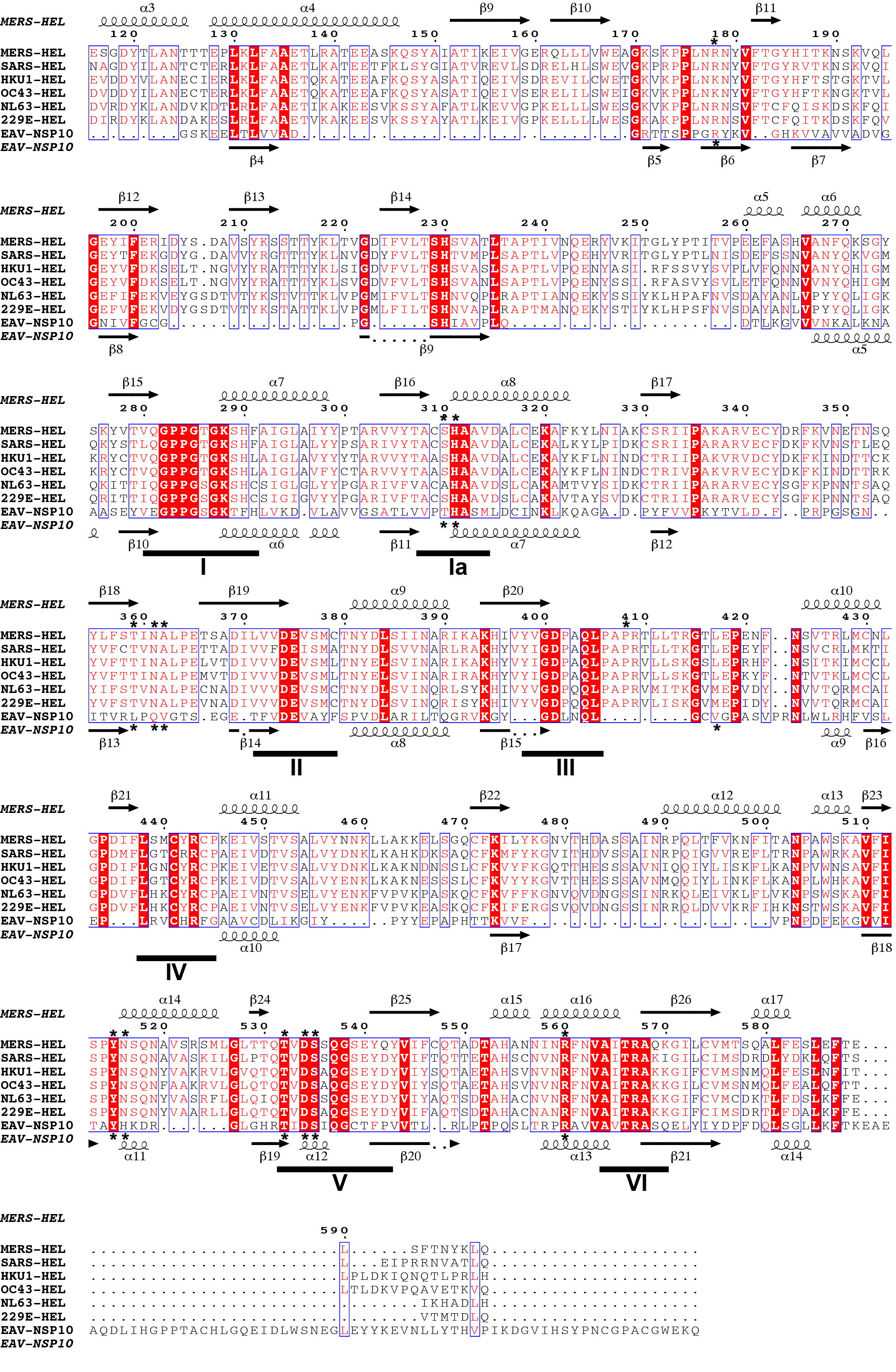

Supplement: S2 Fig — Structure-based multiple sequence alignment of helicase core of nsp13 from human coronavirus (MERS-CoV, SARS-CoV, HKU1-CoV, OC43-CoV, NL63-CoV and 229E-CoV) and EAV nsp10. Sequence alignment of the CH/ZBD domains of CoV nsp13 and EAV nsp10 is shown in Fig 4C. Invariant residues are highlighted with red background; conserved residues are in red. Secondary structure elements are aligned to the top of the sequences. Conserved helicase motifs are indicated at the bottom of the sequences. Multiple sequence alignments were carried out using the program MUSCLE [45]. The program ESPript v3.0 was used to generate the figure[46]. (TIF) [file ppat.1006474.s002.tif]

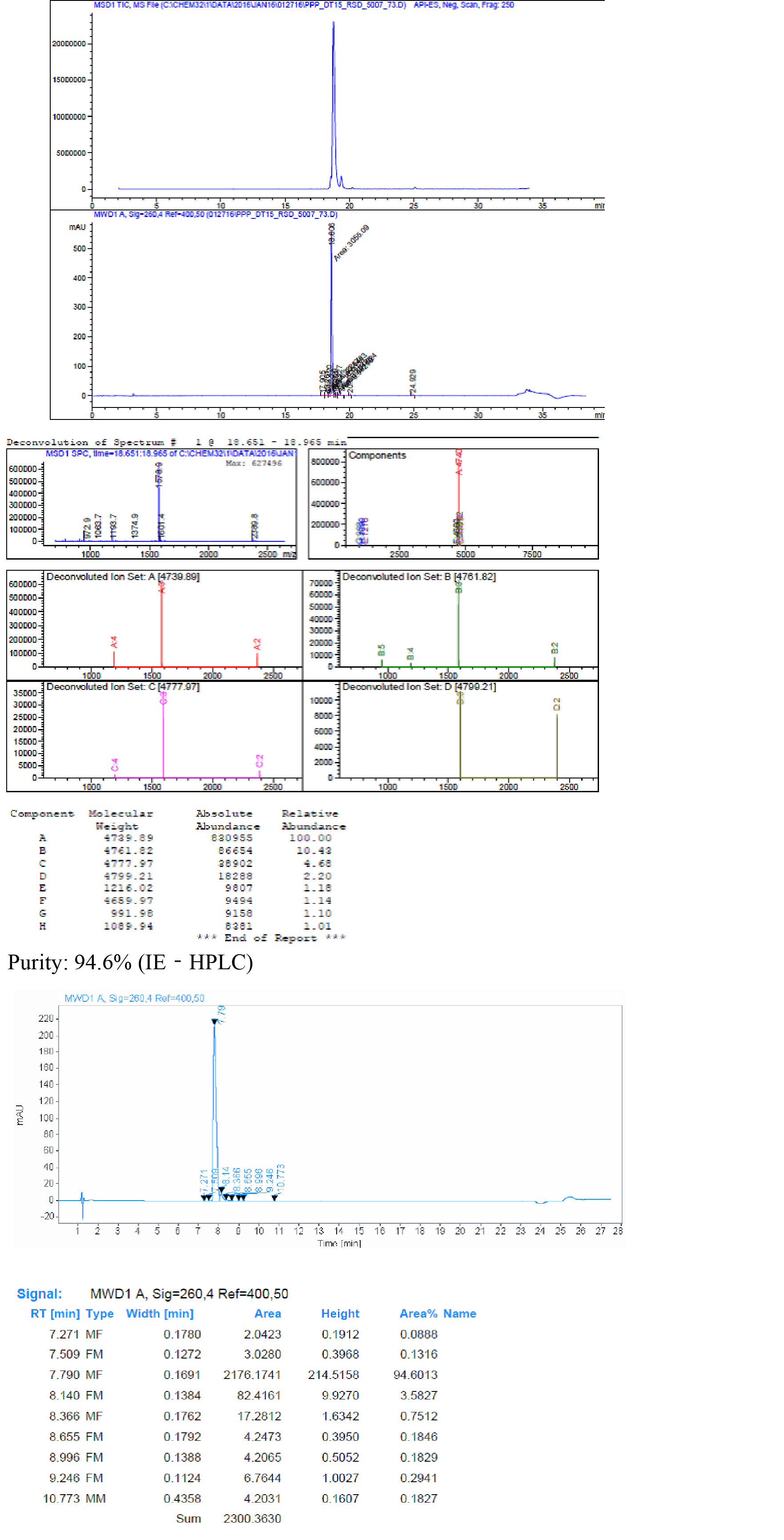

Supplement: S3 Fig — Compound: 5’-PPP-dTdTdTdTdTdTdTdTdTdTdTdTdTdTdT-3’ Calc MW: 4739.64, Found: 4739.89. (TIF) [file ppat.1006474.s003.tif]

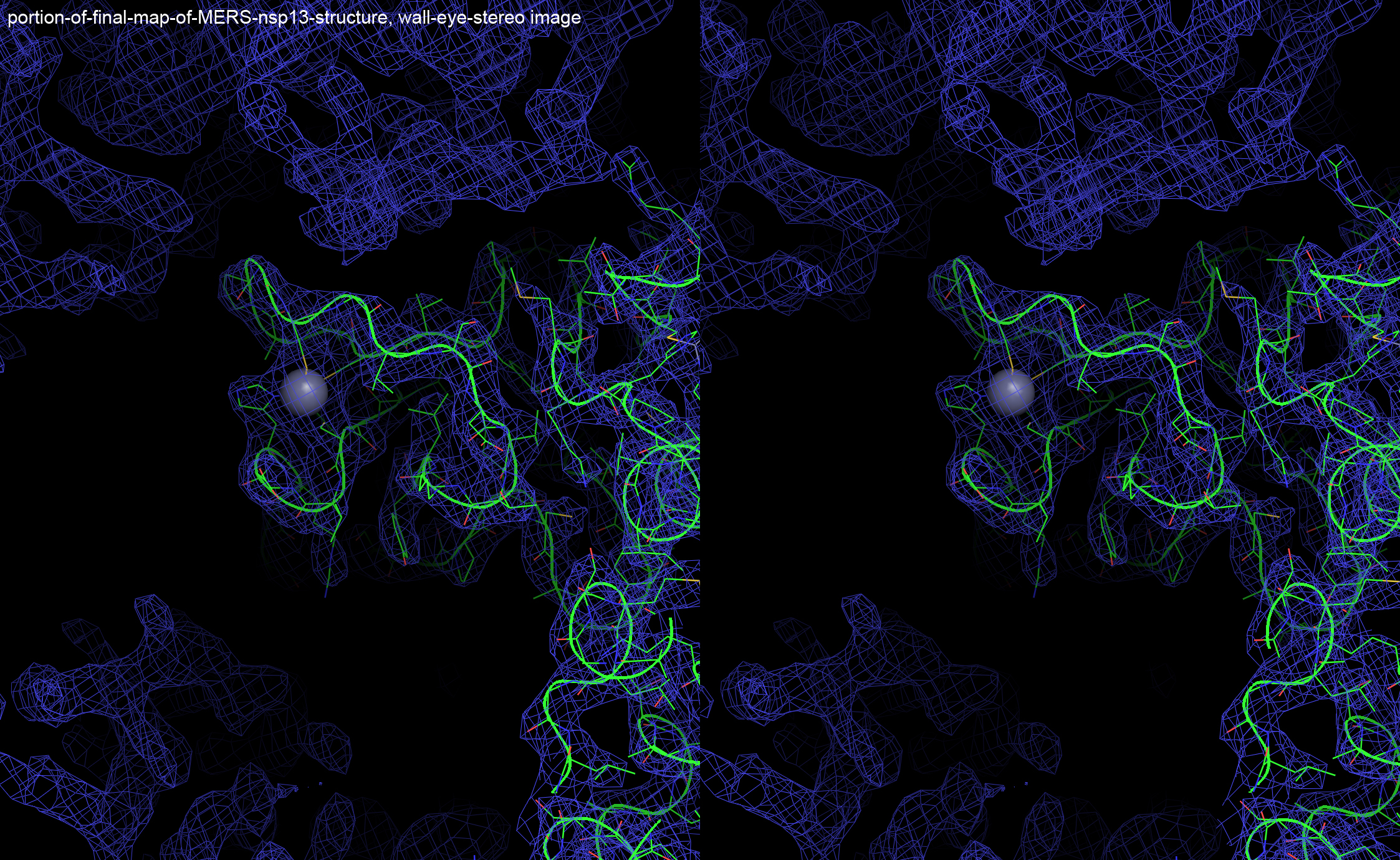

Supplement: S4 Fig — A wall-eye stereo image of a portion of electron density map (zn3 binding site). 2Fo-Fc map is shown with blue mesh. The final model of MERS-CoV nsp13 (green) is superimposed. The zinc is shown with a gray sphere. (JPG) [file ppat.1006474.s004.jpg]
