# Supplementary material for: Crystal structure of Middle East respiratory syndrome coronavirus helicase
Source: PLoS Pathog. 2017 Jun 26;13(6):e1006474. doi: 10.1371/journal.ppat.1006474 (PMC5501694; doi:10.1371/journal.ppat.1006474)
Supplement: S1 Table — (DOCX) [file ppat.1006474.s005.docx]

**Tables S1 Prediction of residues of nsp13 involving in RNA recognition**

|  | **MERS-CoV nsp13** | **EAV nsp10 (4N0O)[**[**1**](#_ENREF_1)**]** | **scUpf1 (2XZL)[**[**2**](#_ENREF_2)**]** | **hUpf1 (2XZO)[**[**2**](#_ENREF_2)**]** |
| --- | --- | --- | --- | --- |
| **1B** | *R178* | R102 |  |  |
| **RecA1** | *S310* | T185 | S461 | S523 |
|  | *H311* | H186 | N461 | N524 |
|  | *T359* | L227 | T553 | T616 |
|  | *N361* | Q229 |  |  |
|  | *A362* | V230 |  |  |
|  | *P408* | V271 | V609 | V665 |
| **RecA2** | *Y515* | Y338 | Y732 | Y796 |
|  | *N516* | H339 | - | - |
|  | *T532* | T348 | - | - |
|  | *D534* | D350 | D763 | D827 |
|  | *S535* | S351 | S761 | S825 |
|  | *R560* | R374 | R794 | R858 |

RNA-binding residues of EAV nsp10 and Upf1 helicases are identified in their crystal structures in complex with RNA. PDB ID of the structures and the related references are indicated.

**References**

1. Deng Z, Lehmann KC, Li X, Feng C, Wang G, et al. (2014) Structural basis for the regulatory function of a complex zinc-binding domain in a replicative arterivirus helicase resembling a nonsense-mediated mRNA decay helicase. Nucleic Acids Res 42: 3464-3477.

2. Chakrabarti S, Jayachandran U, Bonneau F, Fiorini F, Basquin C, et al. (2011) Molecular mechanisms for the RNA-dependent ATPase activity of Upf1 and its regulation by Upf2. Mol Cell 41: 693-703.
